# Rosiglitazone Regulates Anti-Inflammation and Growth Inhibition via PTEN

**DOI:** 10.1155/2014/787924

**Published:** 2014-03-13

**Authors:** Chiou-Feng Lin, Kung-Chia Young, Chyi-Huey Bai, Bu-Chin Yu, Ching-Ting Ma, Yu-Chieh Chien, Chiu-Ling Chiang, Chao-Sheng Liao, Hsin-Wen Lai, Chiung-Wen Tsao

**Affiliations:** ^1^Institute of Clinical Medicine, National Cheng Kung University Medical College, Tainan 70101, Taiwan; ^2^Department of Medical Laboratory Science and Biotechnology, National Cheng Kung University Medical College, Tainan 70101, Taiwan; ^3^School of Public Health, Taipei Medical University, Taipei 11031, Taiwan; ^4^Department of Life Sciences, College of Bioscience and Biotechnology, National Cheng Kung University Medical College, Tainan 70101, Taiwan; ^5^Department of Nursing, Chung Hwa University of Medical Technology, No. 89, Wen-Hwa 1st Street, Jen-Te Hsiang, Tainan 71703, Taiwan; ^6^Department of Gastroenterology, Shin Kong Wu Ho-Su Memorial Hospital, Taipei 11101, Taiwan

## Abstract

Peroxisome proliferator-activated receptor gamma (PPAR*γ*) agonist has anti-inflammatory and anticancer properties. However, the mechanisms by which PPAR*γ* agonist rosiglitazone interferes with inflammation and cancer via phosphatase and tensin homolog-(PTEN)-dependent pathway remain unclear. We found that lower doses (<25 **μ**M) of rosiglitazone significantly inhibited lipopolysaccharide-(LPS)-induced nitric oxide (NO) release (via inducible nitric oxide synthase, iNOS), prostaglandin E2 (PGE_2_) production (via cyclooxygenase-2, COX-2), and activation of Akt in RAW 264.7 murine macrophages. However, rosiglitazone did not inhibit the production of reactive oxygen species (ROS). In PTEN knockdown (shPTEN) cells exposed to LPS, rosiglitazone did not inhibit NO release, PGE_2_ production, and activation of Akt. These cells had elevated basal levels of iNOS, COX-2, and ROS. However, higher doses (25–100 **μ**M) of rosiglitazone, without LPS stimulation, did not block NO release and PGE_2_ productions, but they inhibited p38 MAPK phosphorylation and blocked ROS generation in shPTEN cells. In addition, rosiglitazone caused G1 arrest and reduced the number of cells in S + G2/M phase, leading to growth inhibition. These results indicate that the anti-inflammatory property of rosiglitazone is related to regulation of PTEN independent of inhibition on ROS production. However, rosiglitazone affected the dependence of PTEN-deficient cell growth on ROS.

## 1. Introduction

The thiazolidinedione (TZD) family of drugs is widely used for treatment of type 2 diabetes. TZD activates nuclear regulatory protein peroxisome proliferator-activated receptor gamma (PPAR*γ*) that functions as a transcription factor regulating the expression of various genes and also plays a vital role in modulation of cellular differentiation, development, and metabolism. In addition, TZD inhibits tumorigenesis via regulating cell cycle control, tumor cell recognition of extracellular mitogenic signals, and the expression of angiogenic factors [1–4]. On the other hand, activation of endogenous PPAR*γ* pathway inhibits inflammatory diseases, such as rheumatoid arthritis [5], sepsis [6], and pancreatitis [7]. PPAR*γ* ligands (e.g., 15-deoxy-12,14-PGJ2) and TZD (e.g., rosiglitazone and troglitazone) have been proposed to exert anti-inflammatory effects because they may inhibit phorbol myristyl acetate-induced secretion of proinflammatory cytokines (such as tumor necrosis factor-*α* (TNF-*α*) and interleukin 6 [8–10]) by monocytes and block lipopolysaccharide- (LPS-) induced expressions of the inducible nitric oxide synthase (iNOS) and/or cyclooxygenase-2 (COX-2) [5, 11]. Thus, these agents have a potential application in inflammation treatment [6] and cancer chemoprevention [12].

Phosphatase and tensin homolog deleted on chromosome 10 (PTEN), which encodes a dual-specificity phosphatase, has protein and lipid phosphatase activities that can antagonize PI3K's ability to promote cell proliferation, growth, metabolism, and survival [13]. PTEN was originally identified as a tumor suppressor gene which is frequently mutated or deleted in a variety of human cancers [14]. Persistent PTEN inactivation may provide an etiological link between inflammation and colorectal cancer [15, 16]. Cellular PTEN is vulnerable to inactivation by chemically reactive lipid mediators of inflammation and redox stress [16]. Besides, reactive oxygen species (ROS) act as a double-edged sword controlling tumor cell development and inflammation through the activations of transcription factors (e.g., nuclear factor-*κ*B) and/or the expression of tumor suppressor genes (e.g., PTEN) [17]. However, the precise mechanism underlying this ROS/PTEN pathway needs further investigation.

Activation of PPAR*γ* by rosiglitazone upregulates PTEN expression in inflammatory and tumor-derived cells, including human macrophages, Caco2 colorectal cancer cells, and MCF7 breast cancer cells [18]. Several studies have shown that the anti-inflammatory actions and protective roles of PPAR*γ* agonists involve the upregulation of PTEN, but many of these data are contradictory [9, 19, 20]. Furthermore, regulation of PI3K/Akt and mitogen-activated protein kinase (MAPK) is also involved in alleviating inflammation and/or tumorigenesis by PPAR*γ* ligands and its agonists [21–26]. Taken together, these studies show that PTEN may participate in PPAR*γ* ligand-mediated signaling against inflammation or tumorigenesis through coregulation with Akt and MAPK. Currently, it's not clear whether rosiglitazone would elicit a functional effect in cells with PTEN deficiency.

Myeloid lineage, such as monocytes/macrophages, is one of main sources that produces inflammatory mediators and promotes tumor development. In the present study, we used the lentiviral transduction system to stably express short hairpin RNAs for targeted PTEN knockdown in RAW 264.7 murine macrophages to assess changes in inflammatory response and cell growth, with or without LPS stimulation. Next, we utilized a PTEN-deficient cell line to evaluate the inhibitory effects of rosiglitazone on the above signaling pathways and examined how the generation of ROS affects inflammation and cell growth.

## 2. Materials and Methods

### 2.1. Cell Cultures

RAW 264.7 murine macrophages were purchased from Bioresource Collection and Research Centre (Hsinchu, Taiwan). Cells were maintained (5% CO_2_, 37°C) in Dulbecco's modified Eagle's medium (DMEM; Life Technologies, Rockville, MD) supplemented with 10% heat-inactivated fetal bovine serum (FBS; Invitrogen, Life Technologies, Carlsbad, CA) and 100 *μ*g/mL gentamicin.

### 2.2. Chemical Reagents

*Escherichia coli*-derived LPS was obtained from Calbiochem (San Diego, CA). Rosiglitazone was purchased from Cayman Chemical Co. (Ann Arbor, MI). GW9662 was purchased from Tocris Bioscience Co. (Ellisville, MO).

### 2.3. Antibodies

Mouse anti-*β*-actin monoclonal antibody was purchased from Chemicon International, Inc. (Temecula, CA). Antibodies against phospho-Akt (Ser473), Akt, phospho-p38 MAPK (Thr180/Tyr182), p38 MAPK, phosphoextracellular signal-regulated kinase 1/2 (ERK1/2) (Thr202/Tyr204), ERK1/2, phospho-SAPK/JNK (Thr183/Tyr185), SAPK/JNK, and iNOS were purchased from Cell Signaling Technology, Inc. (Beverly, MA). Rabbit anti-cyclooxygenase-2 (COX-2) polyclonal antibody was purchased from Thermo Fisher Scientific Anatomical Pathology (Cheshire, WA, UK).

### 2.4. Lentiviral-Based RNA Interference Knockdown

PTEN knockdown in RAW 264.7 cells was performed using lentiviral transduction to stably express short hairpin RNAs (shRNAs) that target PTEN. shRNA constructs were purchased from the National RNAi Core Facility (Institute of Molecular Biology/Genomic Research Center, Academia Sinica, Taiwan). Both PTEN shRNA construct (TRCN0000028992, containing the shRNA target sequence 5′-GCTAGAACTTATCAAACCCTT-3′ for mouse PTEN) and the luciferase shRNA construct (TRCN0000072247, containing the shRNA target sequence 5′-GAATCGTCGTATGCAGTGAAA-3′ for a negative control) were used to generate recombinant lentiviral particles. The cells were transduced with the viral particles containing PTEN or luciferase shRNAs for 24 h and then given fresh medium. Cell supernatants were harvested at 36, 48, 60, and 72 h after transduction and then filtered with a 0.45 *μ*m low protein-binding filter. The viral particles were further concentrated by centrifugation at 20,000 ×g at 4°C for 2.5 h and then resuspended with fresh medium. Lentiviral particles (shPTEN and shLuc) were introduced to RAW 264.7 cells with appropriate M.O.I. in medium supplemented with 8 *μ*g/mL polybrene. The knockdown of PTEN was confirmed by Western blotting analysis.

### 2.5. Determination of NO Release

NO production was determined by measuring the production of nitrite in the supernatant with the Griess reagent. Briefly, 100 *μ*L culture supernatant was mixed with 100 *μ*L Griess reagent (1% sulphanilamide, 0.1% naphthylethylenediamine dihydrochloride, and 2.5% H_3_PO_4_) for 10 min at room temperature. The concentration of nitrite was determined spectrophotometrically (Spectra MAX 340PC; Molecular Devices Corporation, Sunnyvale, CA) at 540 nm, and the nitrite concentration was calculated using a standard curve of sodium nitrite with ELISA software (Softmax Pro; Molecular Devices).

### 2.6. Determination of PGE_2_ Production

PGE_2_ production was measured by a PGE_2_ EIA kit (Cayman Chemical Co., Ann Arbor. MI). The concentration of PGE_2_ was measured spectrophotometrically (Spectra MAX 340PC) at 420 nm, and PGE_2_ concentration was calculated using a PGE_2_ EIA standard curve with Softmax Pro ELISA software.

### 2.7. Detection of Intracellular ROS Production

Cells were cultured for indicated times, washed twice with DMEM, and exposed to 20 *μ*M 5-(and-6)-chloromethyl-2′,7′-dichlorodihydrofluorescein diacetate (CM-H_2_DCFDA) (Invitrogen) for 1 h. After washing with PBS, cells were analyzed by flow cytometry using an excitation wavelength of 488 nm (FACSCalibur, BD Biosciences, CA).

### 2.8. Western Blot Analysis

Cells were harvested at the indicated times and lysed with a buffer containing 1% Triton X-100, 50 mM Tris (pH 7.5), 10 mM EDTA, 0.02% NaN_3_, and a protease inhibitor cocktail (Sigma-Aldrich). Protein lysates (40 *μ*g) were separated via 10% SDS-polyacrylamide gel electrophoresis and transferred to a polyvinylidene difluoride membrane (Millipore, Billerica, MA). Membranes were blocked at 25°C for 1 h in TBS-T (10 mM Tris, pH 7.6, 150 mM NaCl, and 0.05% Tween 20) containing 10% skimmed milk and then incubated with 1 : 1000 diluted primary antibodies at 4°C overnight. Subsequently, the blots were washed with TBS-T and incubated with 1 : 5000 diluted horseradish peroxidase-conjugated secondary antibodies at room temperature for 1 h. The protein bands were visualized using an enhanced chemiluminescence kit (PerkinElmer, Boston, MA). For Western blot analysis, *β*-actin was used as the internal control. The optical densities (OD) of phosphoprotein/total protein were determined using the VisionWorks LS software (Upland, CA).

### 2.9. Cell Cycle Analysis by Flow Cytometry

Cells were harvested from culture dishes, washed in phosphate-buffered saline (PBS), suspended in 200 *μ*L ice-cold 70% ethanol, and incubated on ice for at least 1 h. The cells were then washed with PBS, exposed to RNase A (Sigma), and incubated at 37°C for 30 min. The cells were subsequently suspended in propidium iodide (Sigma) in PBS, and DNA analysis was performed using flow cytometry (FACSCalibur). The percentages of cells in sub G1, G0/G1, and S plus G2/M phases were determined.

### 2.10. Cell Proliferation Assay

The rate of cell growth was determined colorimetrically using the 3-2,5-diphenyl tetrazolium bromide (MTT) assay kit (Chemicon International Inc.). Cells (8 × 10^3^ cells per well) were seeded onto 96-well plates and cultured for 24 and 48 h. After cells were incubated with the MTT at 37°C for 4 h, the supernatants were discarded. One hundred microliters of DMSO was added to the cells and allowed to incubate at room temperature for 5 min. Subsequently, absorbance was measured with a Spectra Max 340PC ELISA reader (Molecular Devices) set at an absorbance wavelength of 570 nm.

### 2.11. Statistical Analysis

For the above functional experiments, the results were expressed as means ± standard error of the mean (SEM). Student's *t*-tests were used to analyze the data. Statistical significance was set at *P* < 0.05.

## 3. Results

### 3.1. Rosiglitazone Blocked LPS-Induced NO Release and PGE_2_ Production in RAW 264.7 Cells

**Figure 1:**
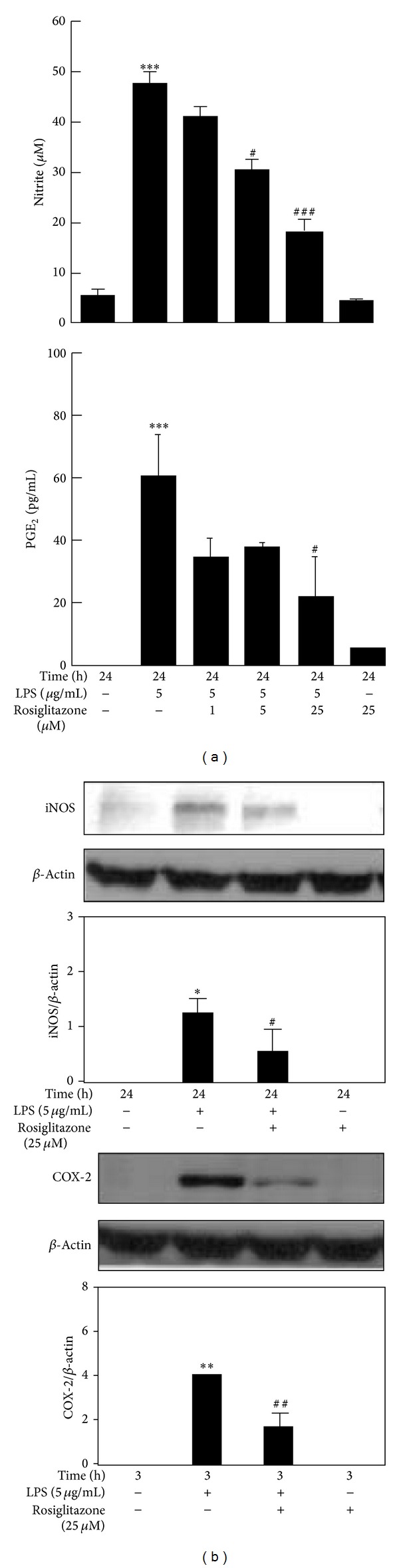
Effects of rosiglitazone on LPS-induced NO release and PGE_2_ production (a) and the expressions of iNOS and COX-2 (b) in RAW 264.7 cells. Cells were treated with 1, 5, or 25 *μ*M rosiglitazone for 30 min before being stimulated with 5 *μ*g/mL LPS for indicated times. Cell supernatants were collected for determining NO release and PGE_2_ production. Cell lysates were harvested for detecting the levels of iNOS, COX-2 and *β*-actin by Western blot. Data shown are representative of three individual experiments. Data are expressed as mean ± SEM obtained from three individual cell cultures. *P < 0.05 and ***P < 0.001 compared with the control, untreated group. ^#^ *P* < 0.05, ^##^ *P* < 0.01, and ^###^ *P* < 0.001 compared with the LPS group.

LPS significantly enhanced NO release and PGE_2_ production at 24 h (Figure 1(a)) and induced the expression of iNOS at 24 h and COX-2 at 3 h posttreatment in RAW 264.7 cells (Figure 1(b)). Pretreatment with rosiglitazone (between 1 and 25 *μ*M) significantly blocked LPS-induced NO release and PGE_2_ production. The levels of expression of iNOS and COX-2 were also reduced in the presence of 25 *μ*M rosiglitazone (Figure 1).

### 3.2. Pretreatment with PPAR*γ* Antagonist Did Not Reverse the Inhibitory Effect of Rosiglitazone on LPS-Induced NO Release and PGE_2_ Production

**Figure 2:**
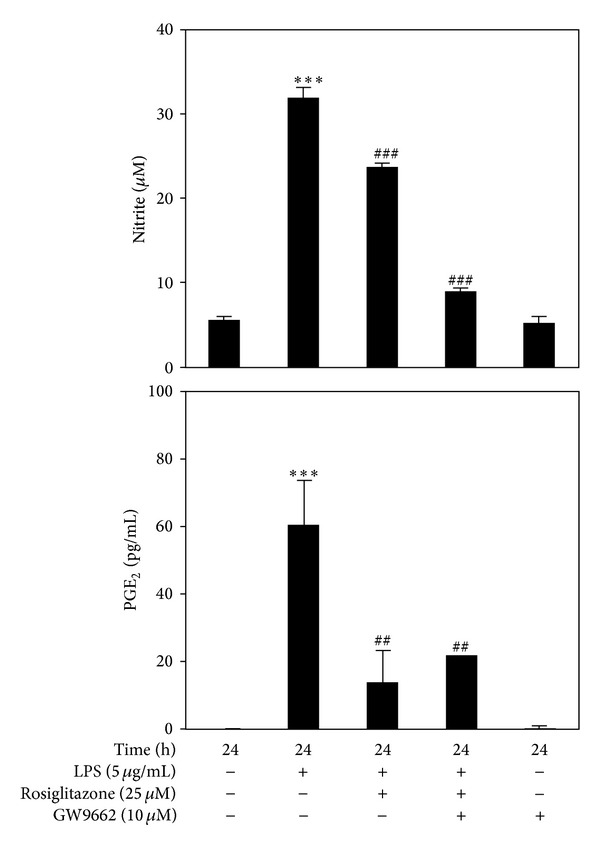
Pretreatment with PPAR*γ* antagonist did not reverse the inhibition of rosiglitazone on LPS-induced NO release and PGE_2_ production. RAW 264.7 cells were treated with 25 *μ*M rosiglitazone for 30 min before being stimulated with 5 *μ*g/mL LPS for 24 h. In some experiments, the cells were first treated with 10 *μ*M GW9662 for 30 min, prior to the addition of 25 *μ*M rosiglitazone for 30 min, and then followed by incubation with 5 *μ*g/mL LPS for 24 h. Cell supernatants were collected for determining NO release and PGE_2_ production. Data shown are representative of three individual experiments. Data are expressed as mean ± SEM obtained from three individual cultures. ***P < 0.001 compared with the control, untreated group. ^##^ *P* < 0.01 and ^###^ *P* < 0.001 compared with the LPS group.

We further investigated the anti-inflammatory effect of rosiglitazone by focusing on PPAR*γ*. PPAR*γ* antagonist GW9622 was added to cells for 30 min before treatment with rosiglitazone. Upon LPS stimulation, GW9622 (10 *μ*M) did not reverse the ability of rosiglitazone to block NO release and PGE_2_ production. This result indicates that anti-inflammatory effect of rosiglitazone is not mediated through PPAR*γ* (Figure 2).

### 3.3. PTEN Knockdown in RAW 264.7 Cells Blocked the Inhibitory Effects of Rosiglitazone on LPS-Induced NO Release and PGE_2_ Production

**Figure 3:**
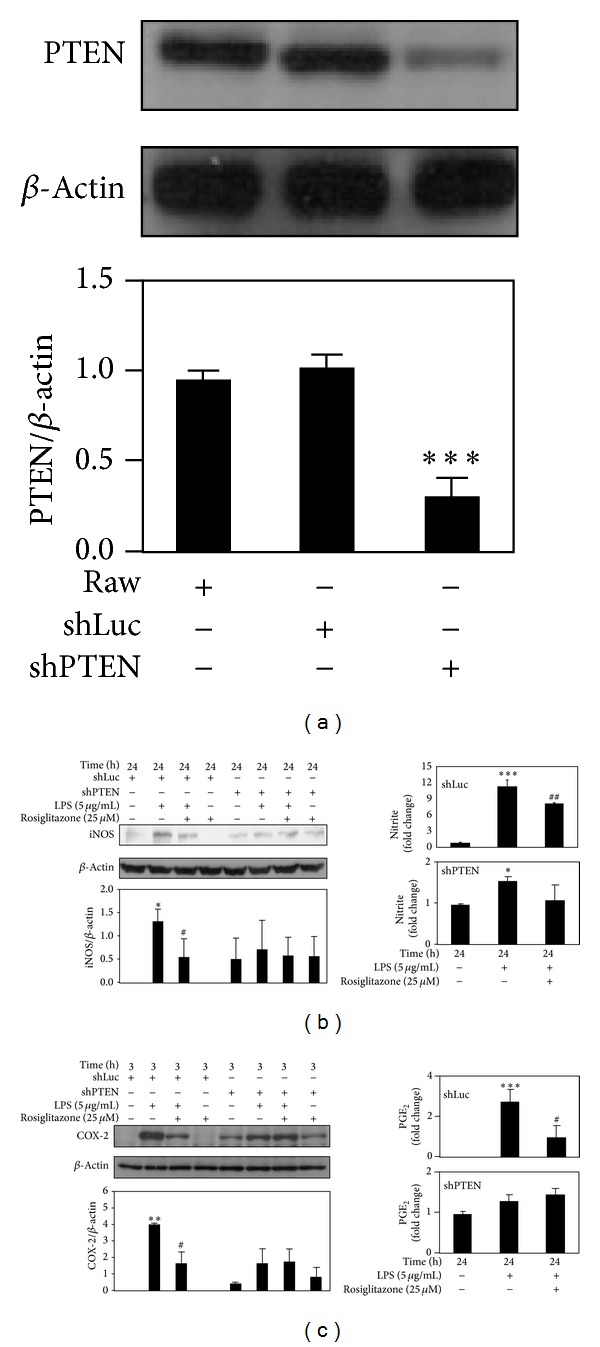
Rosiglitazone did not block LPS-induced iNOS upregulation/NO release and COX-2 upregulation/PGE_2_ production in shPTEN cells. Cells were pretreated with 25 *μ*M rosiglitazone prior to stimulation with 5 *μ*g/mL LPS for indicated times. (a) PTEN protein expression in RAW 264.7 cells transduced with shLuc and shPTEN. Effects of rosiglitazone on the expression of iNOS (b) and COX-2 (c) in shLuc and shPTEN cells. Cell lysates were harvested for detection of levels of PTEN, iNOS, COX-2, and *β*-actin by Western blotting. Cell supernatants were collected for determining NO release and PGE_2_ production. Data shown are representative of three individual experiments. Data are expressed as mean ± SEM obtained from three individual cultures. *P < 0.05, **P < 0.01, and ***P < 0.001 compared with the control, untreated group. ^#^ *P*< 0.05 and ^##^ *P* < 0.01 compared with the LPS group.

RAW 264.7 cells were transduced either with shLuc or shPTEN viral particles. The knockdown of PTEN by shPTEN in RAW 264.7 cells was confirmed by Western blotting (Figure 3(a)). Like untransduced RAW 264.7 cells treated with LPS, similar inhibitory effects of rosiglitazone on NO release and PGE_2_ production were observed in shLuc cells (Figures 3(a) and 3(b)). On the contrary, rosiglitazone at 25 *μ*M did not block LPS-induced expressions of iNOS (Figure 3(a)) and COX-2 (Figure 3(b)) in shPTEN cells.

NO release and PGE_2_ production in shLuc- and shPTEN cells subjected to LPS stimulation were also compared. Upon LPS stimulation, significantly higher production of NO and PGE_2_ was observed in shLuc cells than in shPTEN cells. Compared to the control, untreated cells, LPS markedly increased NO release by 11.5 ± 1.2 fold in shLuc cells, but only by 1.6 ± 0.1 fold in shPTEN cells. Also, PGE_2_ production was increased by 2.8 ± 0.6 fold in shLuc cells, compared to 1.3 ± 0.1 fold in shPTEN cells. Moreover, as shown in Figures 3(b) and 3(c), there was no significant difference in NO release and PGE_2_ production between LPS-treated and rosiglitazone-pretreated shPTEN cells: NO release (1.6 ± 0.1 fold versus 1.1 ± 0.4 fold, *P* = 0.1152) and PGE_2_ production (1.3 ± 0.1 fold versus 1.4 ± 0.2 fold, *P* = 0.2016). Notably, for shPTEN cells, rosiglitazone alone did not inhibit the basal iNOS and COX-2 expression nor NO and PGE_2_ production in the absence of LPS stimulation (Figures 3(b) and 3(c)).

### 3.4. Rosiglitazone Inhibited LPS-Induced Phosphorylation of Akt in shLuc Cells but Not in shPTEN Cells

**Figure 4:**
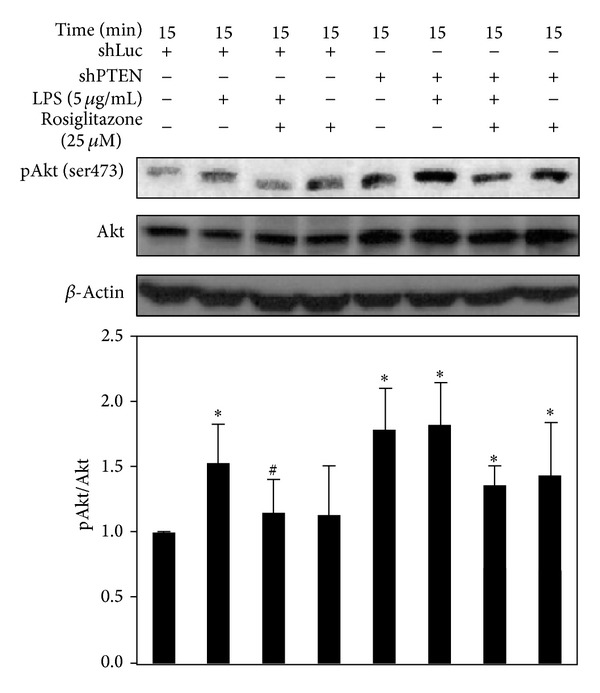
Effects of rosiglitazone on Akt phosphorylation in shLuc and shPTEN cells. Cells were treated with 25 *μ*M rosiglitazone prior to incubation with 5 *μ*g/mL LPS for 15 min. Cell lysates were harvested for determining the levels of phospho-Akt, Akt, and *β*-actin by Western blotting. Data shown are representative of three individual experiments. Data are expressed as mean ± SEM obtained from three individual cultures. *P< 0.05 compared with the control, untreated group. ^#^ *P* < 0.05 compared with the LPS group.

In shLuc cells, LPS increased the phosphorylation of Akt (Ser473) after 15 min. The addition of 25 *μ*M rosiglitazone, however, could inhibit Akt phosphorylation (Figure 4). A similar result was observed in untransduced RAW cells (data not shown). Compared with shLuc cells, shPTEN cells had a higher basal level of Akt phosphorylation. When shPTEN cells were treated with LPS, no further increase in Akt phosphorylation was observed. On the other hand, rosiglitazone slightly, but not significantly, inhibited LPS-induced Akt phosphorylation in shPTEN cells. However, rosiglitazone alone did not inhibit Akt phosphorylation in the absence of LPS stimulation.

### 3.5. Rosiglitazone Did Not Affect LPS-Induced Phosphorylation of MAPK Family of Proteins and ROS Generation in shLuc and shPTEN Cells

**Figure 5:**
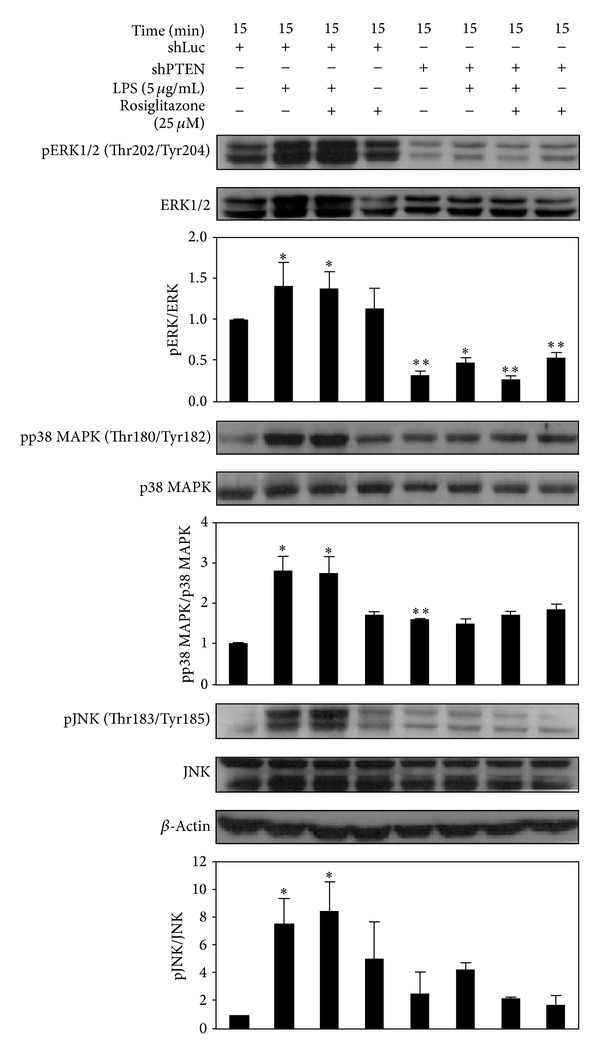
Effects of rosiglitazone on phosphorylation of MAPK family of proteins in shLuc and shPTEN cells. Cells were treated with 25 *μ*M rosiglitazone prior to stimulation with 5 *μ*g/mL LPS for 15 min. Cell lysates were prepared for determining the levels of phospho-ERK1/2, ERK1/2, phospho-p38 MAPK, p38 MAPK, phospho-JNK, JNK, and *β*-actin by Western blotting. Data shown are representative of three individual experiments. Data are expressed as mean ± SEM obtained from three individual cultures. *P < 0.05 and **P < 0.01 compared with the control, untreated group.

In addition to activating Akt, LPS increased the phosphorylation of p38 MAPK (Thr180/Tyr182), ERK1/2 (Thr202/Tyr204), and JNK (Thr183/Tyr185) after 15 min in shLuc cells. On the other hand, rosiglitazone alone (25 *μ*M) did not affect the phosphorylation levels of the MAPK family of proteins (Figure 5). When shPTEN cells were treated with LPS, no significant increase in the phosphorylation of the MAPK family of proteins was observed. Similarly, rosiglitazone did not have any effect on the phosphorylation of these proteins upon LPS stimulation (Figure 5). Knockdown of PTEN appeared to decrease the basal level of phospho-ERK1/2 while increasing phospho-p38 MAPK.

**Figure 6:**
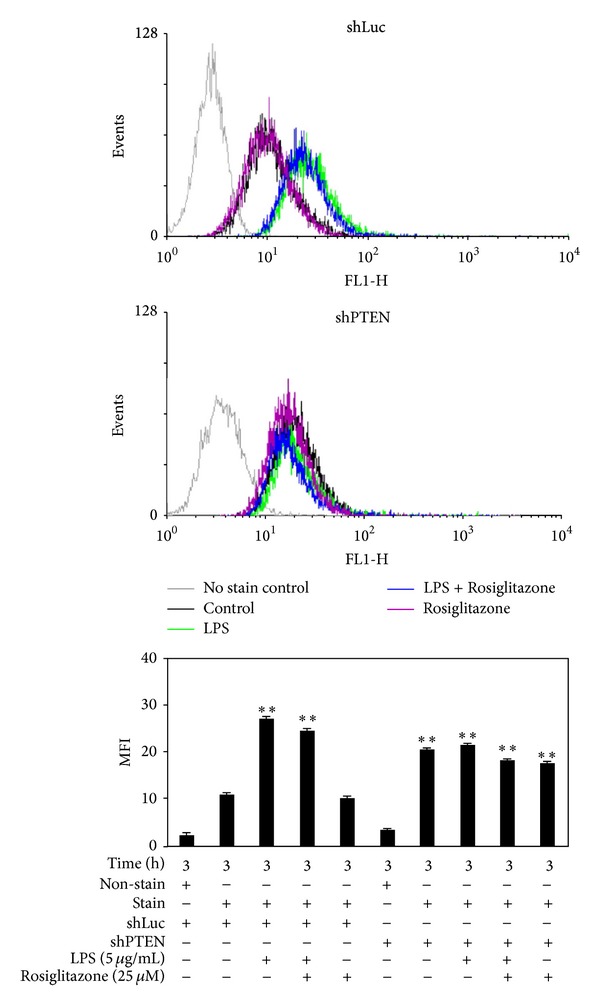
Effects of rosiglitazone on ROS generation in shLuc and shPTEN cells. Cells were treated with 25 *μ*M rosiglitazone prior to stimulation with 5 *μ*g/mL LPS for 3 h. Cell were harvested, stained with DCFH-DA, and then analyzed by FACScan. Data shown are representative of three individual experiments. Data are expressed as mean ± SEM obtained from three individual cultures. MFI stands for mean fluorescence intensity. **P < 0.01 compared with the control, untreated shLuc cells.

Flow cytometry analysis indicated that LPS stimulated ROS generation, whereas rosiglitazone slightly, but not significantly, inhibited ROS generation in shLuc cells after 3 h (Figure 6) and 24 h (data not shown) of treatment. Compared with shLuc cells, shPTEN cells had a significantly higher basal level of ROS. In shPTEN cells, LPS treatment did not lead to higher production of ROS. Rosiglitazone treatment also did not affect ROS production in shPTEN cells with or without LPS stimulation.

### 3.6. Effect of Higher Doses of Rosiglitazone on NO Release, PGE_2_ Production, and p38 MAPK, Akt, ERK1/2, and JNK Phosphorylation in shPTEN Cells

**Figure 7:**
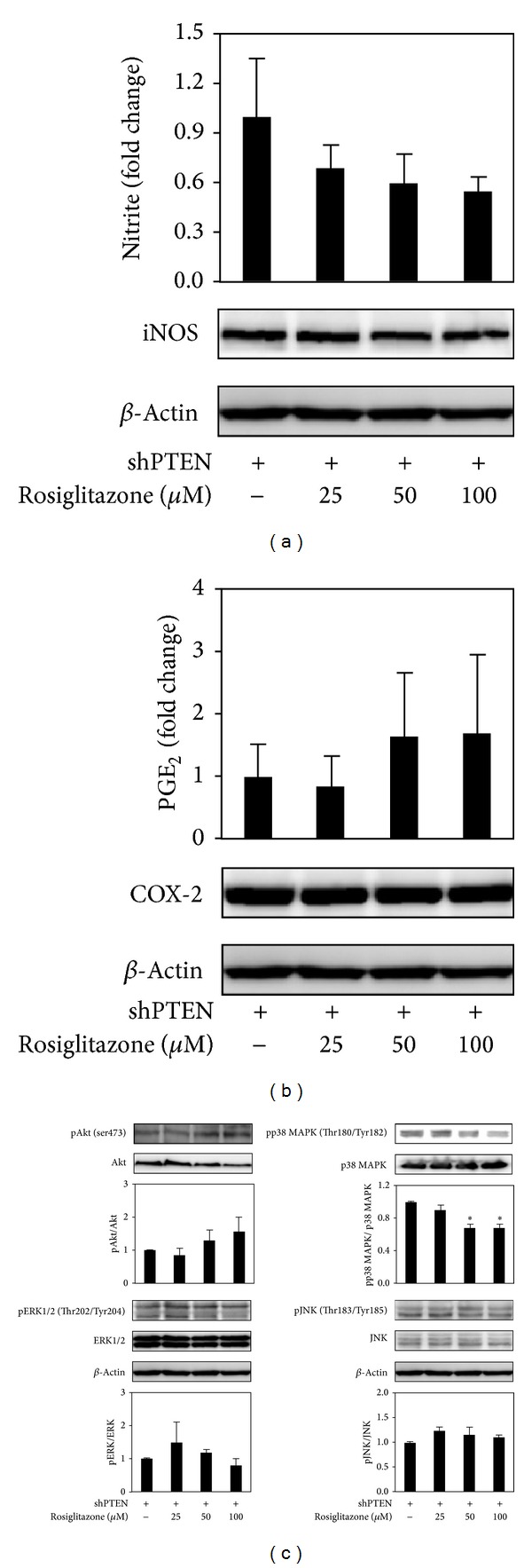
Effects of higher doses (>25 *μ*M) of rosiglitazone on inflammatory response in shPTEN cells. Cells were treated with higher doses (>25 *μ*M) of rosiglitazone and NO release (a), PGE_2_ production (b), and activations of Akt and MAPK family of proteins (c) were determined. Cells were treated with rosiglitazone for 24 h. Cell supernatants were collected for measuring NO release and PGE_2_ production. Cell lysates were harvested for determining the levels of iNOS, COX-2, phospho-Akt, Akt, phospho-p38 MAPK, p38 MAPK, phospho-ERK1/2, ERK1/2, and phospho-JNK, JNK, and *β*-actin by Western blotting. Data shown are representative of three individual experiments. Data are expressed as mean ± SEM obtained from three individual cultures. *P < 0.05 compared with the control, untreated group.

For shPTEN cells, exposure to 25 *μ*M rosiglitazone did not significantly reduce NO release (Figure 7(a)) and PGE_2_ production (Figure 7(b)). However, higher doses of rosiglitazone at 50 and 100 *μ*M did not further affect the reduction of NO release and PGE_2_ production. In addition, protein expression of iNOS and COX-2 was no changed by higher doses of rosiglitazone. Furthermore, higher doses of rosiglitazone appeared to decrease the phosphorylation of p38 MAPK. They did not significantly affect the phosphorylation of Akt, ERK1/2, and JNK (Figure 7(c)).

### 3.7. Higher Doses of Rosiglitazone Inhibited ROS Generation and Cell Growth in shPTEN Cells

**Figure 8:**
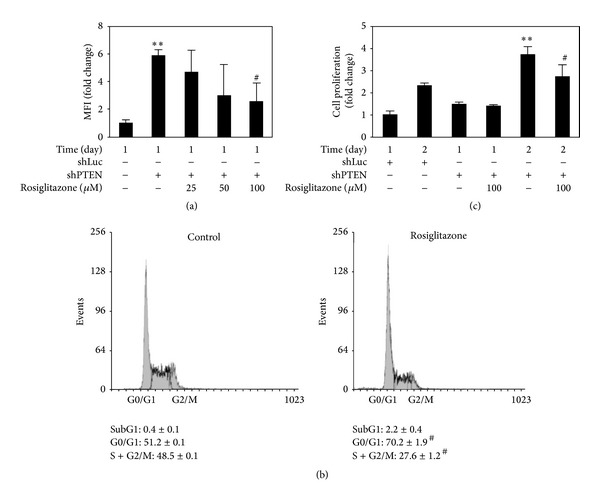
Higher doses of rosiglitazone inhibited ROS generation and cell growth in shPTEN cells. Rosiglitazone affected ROS generation (a), cell cycle phase distribution (b), and cell proliferation (c). shLuc and shPTEN cells were treated with 25–100 *μ*M rosiglitazone for indicated times. Then, cells were harvested and stained with either DCFH-DA or propidium iodide and analyzed by flow cytometry. Cell growth was determined with the MTT assay kit. Data are expressed as means ± SEM obtained from three individual cultures. MFI stands for mean fluorescence intensity. **P < 0.01 compared with the shLuc cells. ^#^ *P* < 0.05 compared with the shPTEN cells.

Flow cytometry analysis revealed that rosiglitazone at 100 *μ*M inhibited markedly ROS generation after one day of treatment (Figure 8(a)). Cell cycle analysis was also carried out by measuring the percentages of cells in sub G1-, G0/G1-, and S plus G2/M-phases. Rosiglitazone was found to cause G1 arrest to 70.2 ± 1.9% of the cells compared to 51.2 ± 0.1% for control, untreated cells. A lower percentage of the treated cells were found to exist in S plus G2/M phase (27.6 ± 1.2%) compared to the control, untreated cells (48.5 ± 0.1%; Figure 8(b)). Cell proliferation was assessed by the MTT assay. The results indicated that shPTEN cells had a significant higher rate of proliferation compared to shLuc cells. Addition of rosiglitazone (100 *μ*M) appeared to reduce cell proliferation by 36.4% by Day 2 (Figure 8(c)).

## 4. Discussion

**Figure 9:**
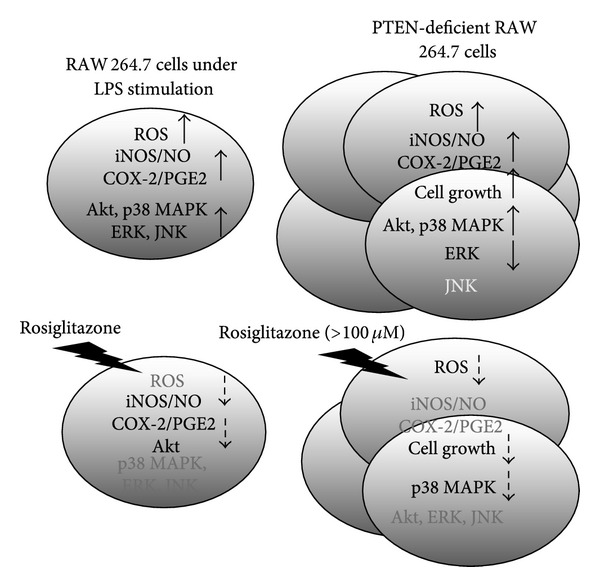
A schematic model of the anti-inflammatory and growth inhibitory effects of rosiglitazone in PTEN-deficient RAW 264.7 cells. Lower doses (<25 *μ*M) of rosiglitazone inhibited LPS-induced NO release, PGE_2_ production, and activation of Akt in RAW 264.7 murine macrophages. These anti-inflammatory effects of rosiglitazone were dependent on PTEN, but they did not affect the production of ROS. In addition to a significantly higher inflammatory response and elevated production of ROS, PTEN knockdown caused increased cell growth and altered signaling molecule expression including Akt, p38 MAPK, and ERK. On the other hand, higher doses (<100 *μ*M) of rosiglitazone delayed cell growth inhibition in PTEN-deficient cells through inhibition of ROS production and p38 MAPK activation.

In our study, LPS significantly induced NO release and PGE_2_ production in RAW 264.7 cells, and these processes were inhibited by rosiglitazone. In addition, the anti-inflammatory effect of rosiglitazone appeared to involve the inactivation of Akt that was independent of PPAR*γ*. These results are consistent with previous studies utilizing PPAR*γ* ligands in primary rat astrocytes, osteoblast-like cells MC3T3E-1, and RAW 264.7 cells [11, 27, 28]. Moreover, we have demonstrated that PTEN-deficient cells had higher basal ROS production, elevated inflammatory mediator secretion, and increased tumor cell growth, as well as enhanced activation of Akt and p38 MAPK and decreased activation of ERK1/2. Furthermore, rosiglitazone caused growth inhibition by promoting G1 arrest and decreasing cells in S plus G2/M phases via inhibiting p38 MAPK activation and ROS production. However, rosiglitazone did not alter the enhanced production of NO and PGE_2_ in PTEN-deficient cells (Figure 9).

In the pathogenesis of cancer and inflammation, ROS acts as a double-edged sword to regulate signaling molecules [17]. A previous study has reported that activation of PPAR*γ* nuclear receptor by synthetic 15-J2-cyclopentenone isoprostanes could markedly inhibit iNOS and COX-2 expression via a partial redox-dependent mechanism [28]. However, we found that the anti-inflammatory effect of rosiglitazone on LPS-stimulated RAW 264.7 cells was not modulated by inhibition of ROS production. This discrepancy may be due to different stimulators being used and different assay time points. Besides, PTEN also controls inflammation and cancer, especially in the liver diseases [29]. This regulation appears to involve ROS modulation of the proapoptotic and pro-cell survival proteins [30, 31]. Endogenously produced ROS has been shown to inactivate PTEN in a macrophage cell line [32]. The ROS-mediated breast cancer cell proliferation was found to be related to the activation of PI3K pathway and reduction of PTEN activity [33]. PTEN inactivation by alkylation of PTEN activates Akt in HEK-293 human embryonic kidney cells [34]. In our study, higher basal ROS production and activation of Akt were observed in PTEN-deficient cells to promote inflammation and cell growth. However, rosiglitazone did not block the increased levels of NO and PGE_2_ in PTEN-deficient cells, whereas high dosage of rosiglitazone inhibited ROS generation to reduce cell growth. Our results implied that ROS could serve different functions including PTEN-regulated anti-inflammatory property and growth inhibition by rosiglitazone.

We have also pointed out that PTEN-deficient cells without extrinsic inflammatory stimulation could develop augmented inflammatory response and ROS production to accelerate cell growth. This likely involved altered activation of signaling molecules including Akt, p38 MAPK, and ERK1/2. Our results were consistent in part with a previous study which showed that PTEN-deficient human mast cells displayed constitutive activation of Akt, p38 MAPK, and JNK to promote cytokine secretion and cell survival [35]. Hepatic steatosis, inflammation, and even carcinogenesis were observed in PTEN-deficient mice [36]. PTEN deficiency has been shown to result in steatohepatitis and hepatic tumorigenesis due to ROS generation and abnormal activations of PKB/Akt and ERK1/2 [37]. Compared to the control shLuc cells, PTEN knockdown caused reduced inflammation response to LPS induction and enhanced activation of Akt (Figures 3 and 4). Similarly, a previous study had shown that PTEN-deficient cells had reduced inflammatory cytokine production in response to gram-negative bacteria and LPS [38]. In our study, we have shown that LPS-induced ROS production was observed in shLuc cells but not in shPTEN cells (Figure 6). Though the precise mechanisms are not understood, this phenomenon may be explained by the fact that the shPTEN cells are able to produce continuously higher levels of ROS, which could cause a resistance to extrinsic stimuli that promote ROS generation. Thus, PTEN knockdown attenuates inflammatory responses and enhances tumor cell survival* in vivo* and* in vitro*.

Several studies have indicated that alteration in PI3K/Akt and MAPK signaling by PPAR*γ* ligands and its agonists may be responsible for the diminished inflammation and tumorigenesis. Adenovirally expressed PPAR*γ* has been shown to exert inhibitory effect against LPS-induced inflammatory response via suppression of ERK activation in human dental pulp cells [39]. On the other hand, pioglitazone exerted inhibitory effect against LPS-induced inflammatory response in dopaminergic neurons [23] and in microglia [24] via inhibition of p38 MAPK activity. PPAR*γ* ligands activate ERK1/2 in cancer cell lines, which is associated with antineoplastic actions [25]. A PPAR*γ* ligand 15d-PGJ2 induces apoptosis via increased inhibition of ERK and decreased inhibition of p38 MAPK in MIA PaCa-2 human pancreatic cells [40]. In our study, rosiglitazone at 25 *μ*M significantly blocked LPS-induced production of NO and PGE_2_, which was related to inhibition of Akt activation in shLuc (Figure 3). Rosiglitazone did not appear to inhibit LPS-induced activation of Akt in shPTEN cells (Figure 4). Rosiglitazone alone did not inhibit the inflammatory response without LPS stimulation. Furthermore, rosiglitazone at >25 *μ*M dose-dependently inhibited the activation of p38 MAPK, whereas it did not significantly affect activations of Akt, ERK1/2, and JNK (Figure 7).

Anti-inflammatory actions and protective roles of PPAR*γ* agonists are also mediated via upregulation of PTEN in cigarette smoke-induced mucin production [19] and asthmatic inflammation [20]. Conversely, PPAR*γ*-dependent anti-inflammatory action of rosiglitazone suppresses TNF-*α* secretion and is not mediated by PTEN in human monocytes [9]. We found that anti-inflammatory actions of rosiglitazone were modulated via upregulation of PTEN in LPS-induced inflammation (Supplementary figure 1 in Supplementary Material available online at http://dx.doi.org/10.1155/2014/787924). However, in our PTEN-deficient cell model, higher dosages of rosiglitazone did not decrease inflammatory response nor activation of Akt, thereby indicating that the anti-inflammatory property of PPAR*γ* agonist rosiglitazone is dependent on PTEN. Moreover, our results also indicate that PTEN-deficient cells could counteract anti-inflammatory responses to rosiglitazone because they had a higher basal inflammatory environment. However, the detailed mechanisms required further investigation.

Several studies with cell and animal models have reported that activation of PPAR*γ* could attenuate cellular stress [41, 42] and inflammation [39, 43] by various stimuli and is concomitant with a reduction of ROS generation. In our study, higher doses of rosiglitazone also inhibited growth inhibition of PTEN-deficient cells via inhibition of ROS production (Figure 8). PPAR*γ* upregulates PTEN expression, which is involved in the inhibition of cell growth and the induction of cell apoptosis in various cancer cells, including hepatocellular carcinoma [44, 45], colon cancer [46], and non-small-cell lung cancer cells (NSCLC) [47]. In addition to PTEN, PPAR*γ* agonist rosiglitazone also downregulates Akt/mTOR/p70S6K signal cascade, which inhibits NSCLC cell proliferation through PPAR*γ*-dependent and PPAR*γ*-independent signaling [48]. However, PPAR*γ* agonists also diminish activation of Akt-1 in 3T3-L1 adipocyte apoptosis without affecting PI3K and PTEN [49]. Rosiglitazone-induced PTEN expression is accompanied by a decline in activations of Akt and MAPK and a rise in G1 arrest in MCF-7 cells [26, 50]. In our study, rosiglitazone caused G1 arrest and lowered proportion of cells in S+G2M phase, which resulted in growth inhibition in PTEN-deficient cells. Blockade of ROS generation and inactivation of p38 MAPK were also involved. However, this process was independent of the activations of Akt. Accordingly, the possible mechanisms of interregulation between ROS and p38 MAPK following rosiglitazone are still unclear, though they may play important roles in cell growth in the absence of PTEN.

## 5. Conclusion

Taken together, these results indicate that the anti-inflammatory property of rosiglitazone was correlated with the regulation of PTEN. But this effect was independent of the inhibition of ROS. Moreover, rosiglitazone affected PTEN-deficient cell growth through inhibition of the activation of p38 MAPK and production of ROS.

## Supplementary Material

Supplementary figure 1: Rosiglitazone affects Akt activation and PTEN expression. Cells were treated with 25 microM rosiglitazone prior to incubation with 5 microg/ml LPS for 60 min. Cell lysates were harvested for determining the levels of phospho-Akt, Akt, PTEN, and beta-actin by Western blotting. Data shown are representative of three individual experiments. Data are expressed as mean ± SEM obtained from three individual cultures.
