# Supplementary material for: Rosiglitazone Regulates Anti-Inflammation and Growth Inhibition via PTEN
Source: Biomed Res Int. 2014 Mar 13;2014:787924. doi: 10.1155/2014/787924 (PMC3971553; doi:10.1155/2014/787924)
Supplement: Supplementary file 1 — Supplementary figure 1: Rosiglitazone affects Akt activation and PTEN expression. Cells were treated with 25 microM rosiglitazone prior to incubation with 5 microg/ml LPS for 60 min. Cell lysates were harvested for determining the levels of phospho-Akt, Akt, PTEN, and beta-actin by Western blotting. Data shown are representative of three individual experiments. Data are expressed as mean ± SEM obtained from three individual cultures. [file 787924.f1.pdf]

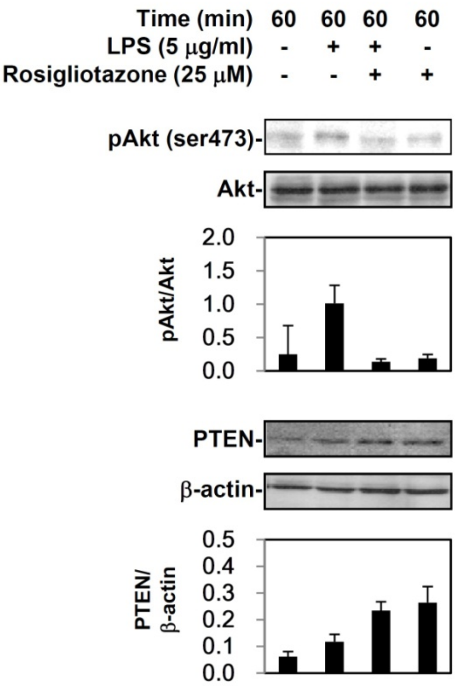

Supplementary figure 1 Rosiglitazone affects PTEN expression. Cells were treated with 25 μM rosiglitazone prior to incubation with 5 μg/ml LPS for 60 min. Cell lysates were harvested for determining the levels of phospho-Akt, Akt, PTEN, and β-actin by Western blotting. Data shown are representative of three individual experiments. Data are expressed as mean ± SEM obtained from three individual cultures.

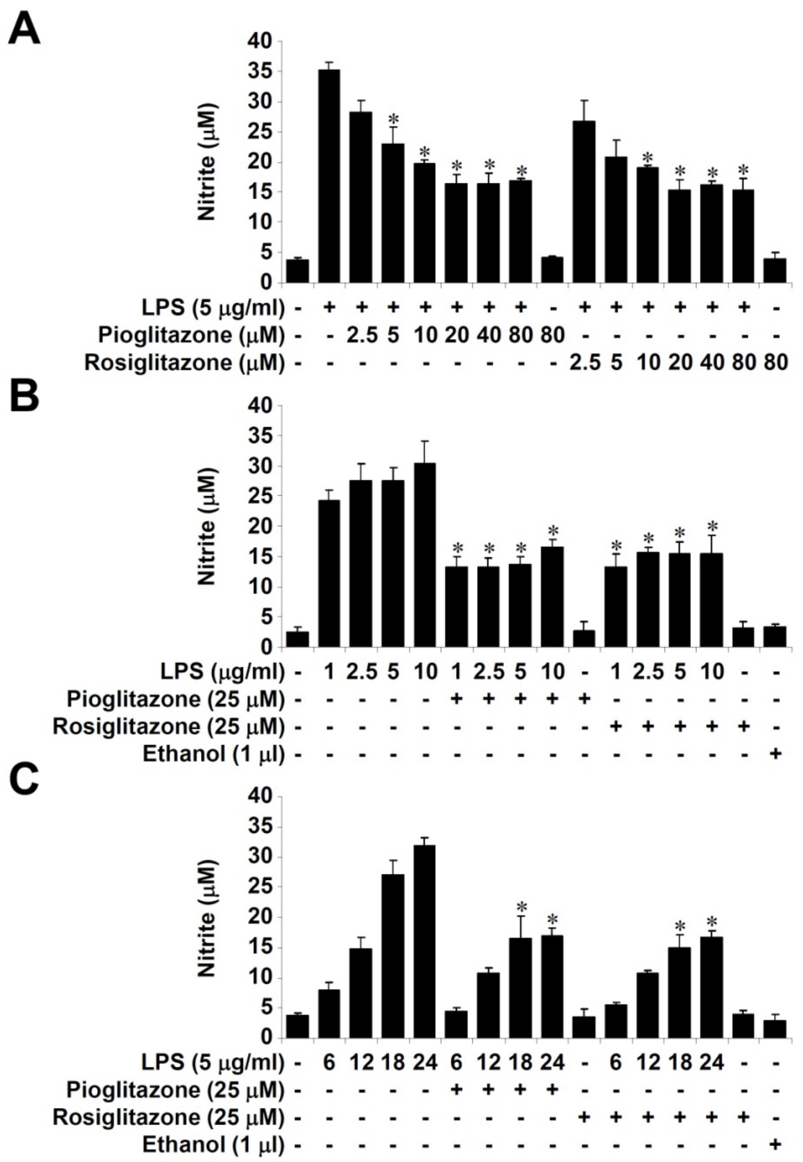

Supplementary figure 2 Effects of pioglitazone and rosiglitazone on LPS-induced NO release (A-C). Cells were treated with 2.5~80 μM pioglitazone or rosiglitazone for 30 min before being stimulated with 1~10 μg/ml LPS for indicated times. Cell supernatants were collected for determining NO release. Data are expressed as mean ± SEM obtained from three individual cell cultures. *P < 0.05 compared with the LPS group.
